# Effects of Manure Compost Application on Soil Microbial Community Diversity and Soil Microenvironments in a Temperate Cropland in China

**DOI:** 10.1371/journal.pone.0108555

**Published:** 2014-10-10

**Authors:** Zhen Zhen, Haitao Liu, Na Wang, Liyue Guo, Jie Meng, Na Ding, Guanglei Wu, Gaoming Jiang

**Affiliations:** 1 State Key Laboratory of Vegetation and Environmental Change, Institute of Botany, the Chinese Academy of Sciences, Beijing, China; 2 Laboratory of Crop, Guangdong Ocean University, Zhanjiang, China; 3 University of the Chinese Academy of Sciences, Beijing, China; 4 Development and Promotion Center, Shandong Small and Medium-sized Enterprises, Ji'nan, China; 5 State Key Laboratory of Crop Biology, Shandong Agricultural University, Tai'an, China,; Agroecological Institute, China

## Abstract

The long-term application of excessive chemical fertilizers has resulted in the degeneration of soil quality parameters such as soil microbial biomass, communities, and nutrient content, which in turn affects crop health, productivity, and soil sustainable productivity. The objective of this study was to develop a rapid and efficient solution for rehabilitating degraded cropland soils by precisely quantifying soil quality parameters through the application of manure compost and bacteria fertilizers or its combination during maize growth. We investigated dynamic impacts on soil microbial count, biomass, basal respiration, community structure diversity, and enzyme activity using six different treatments [no fertilizer (CK), N fertilizer (N), N fertilizer + bacterial fertilizer (NB), manure compost (M), manure compost + bacterial fertilizer (MB), and bacterial fertilizer (B)] in the plowed layer (0–20 cm) of potted soil during various maize growth stages in a temperate cropland of eastern China. Denaturing gradient electrophoresis (DGGE) fingerprinting analysis showed that the structure and composition of bacterial and fungi communities in the six fertilizer treatments varied at different levels. The Shannon index of bacterial and fungi communities displayed the highest value in the MB treatments and the lowest in the N treatment at the maize mature stage. Changes in soil microorganism community structure and diversity after different fertilizer treatments resulted in different microbial properties. Adding manure compost significantly increased the amount of cultivable microorganisms and microbial biomass, thus enhancing soil respiration and enzyme activities (*p*<0.01), whereas N treatment showed the opposite results (*p*<0.01). However, B and NB treatments minimally increased the amount of cultivable microorganisms and microbial biomass, with no obvious influence on community structure and soil enzymes. Our findings indicate that the application of manure compost plus bacterial fertilizers can immediately improve the microbial community structure and diversity of degraded cropland soils.

## Introduction

Chemical fertilizers have been extensively applied to sustain global agricultural production since the first Green Revolution [1], [2]. Modern high-intensity agricultural ecosystems are characterized by the excessive use of chemical fertilizers, pesticides, and herbicides [1]. Chemical fertilizers directly enhance crop yield because plants directly or indirectly assimilate the nutrients provided by these inorganic fertilizers. However, on one hand, the production and use of these chemicals impart various negative effects on the agricultural ecosystem such as degradation of the soil, loss of crop genetic diversity, reduction in soil microbial diversity, contamination of ground-water resources, and pollution of the atmosphere [3], [4]. On the other hand, with the intensive development of animal husbandry, animal dung has become one of the major pollution sources in China. There are a lot of organic matter and nutrients such as nitrogen and phosphorus in animal manure, especially through composting. Application of manure compost may also enhance soil microbial activities that improve the crop growth, and restrain the pests and diseases Compared with chemical fertilizers, manure compost has been comprehensively tested and determined as effective in increasing nutrient availability to crops, thus improving grain yield in a cost-effective and environmentally friendly manner [5], [6]. The addition of manure compost can also increase the levels of organic matter and improve soil porosity, structural stability, moisture, and nutrient availability, as well as biological activity [7], [8]. Hence, it is becoming a more popular practice to add manure compost to the soils if the degraded cropland is considered to be restored.

Soil microorganisms play important roles in ecological functions such as nutrient cycling and formation of soil aggregates through the decomposition of organic matter [9]. The stability of the microbial community structure has important implications for the rates of soil processes. For example, variations in microbial community structure in the soil influence rates of de-nitrification, nitrification, and nitrogen fixation [10], [11]. Organic and inorganic amendments can significantly affect soil microorganisms [12]. Changes in microbial activity and composition, for instance, can influence plant growth by enhancing nutrient turnover and suppressing or mitigating disease incidence [13]. In addition, soil microbial biomass, activity, and community structure are useful indicators of soil quality and health because these parameters are sensitive to changes in cropland management practices [14]. Thus, the adaption of soil microbial structure and functions to the environment are considered essential for sustainable agricultural production [15]. Nevertheless, methods of increasing the soil microbiological diversity and/or restoring the microorganism communities when the soils have been seriously degraded after the long-term application of chemical fertilizers remain largely unknown.

Previous studies have focused on the influence of fertilizers on soil microbial communities, resulting in positive or neutral effects. Some studies have reported that soils in organic farming regimes had higher microbial functional diversity than those in conventional farming systems [16]. According to some investigators, the bacterial diversity was always higher in manure compost-amended farmyard soils regardless of land use patterns or seasons [17]. Although fertilization has resulted in increases in crop yield, this application was not sufficient in triggering a significant improvement in the soil microbial properties [8]. Even some argued [18] that there were no significant differences in bacterial communities between improved and unimproved grassland [18]. While numerous studies have investigated the effects of organic fertilization on microbial communities [19], [20], the dynamic patterns of bacteria, fungi, and actinomycetes under different types of fertilization have remained elusive.

Though some studies have recorded the long-term effects of different fertilization practices on soil microbial properties, few attempts have been made to improve the degraded farmland by enhancing soil microbial properties, especially under conditions that do not include cropland resting. Understanding how soil microbial properties respond to manure compost may help to strengthen agricultural ecosystem health practices. In this study, we hypothesized that adding manure compost and bacterial fertilizers to the soils that have been experienced with chemical fertilizers for several decades may rapidly improve soil microbial diversity. Our objectives were: (i) to identify a more effective method in rapidly improving degraded cropland soil quality through fertilizer management; (ii) to analyze the bacterial and fungal community structure and their growth stage-related dynamics in degraded and restored soils.

## Materials and Methods

### Site description

The study was conducted at the Agricultural Ecosystem Research Station of Shandong Agricultural University in Jiang Jiazhuang Village, Pingyi County, Shandong Province of Eastern China (35°26′21″N, 117°50′11″E). The farm area is in a typical temperate and monsoonal climate, with a mean annual rainfall of 770.2 mm, and an average annual temperature of 13.2°C.

### Experimental design

Six treatments using different fertilizer applications were designed. Fertilizer treatments were established as follows: no fertilizer (CK), N fertilizer (N), N fertilizer + bacterial fertilizer (NB), manure compost (M), manure compost + bacterial fertilizer (MB), and bacterial fertilizer (B). The experimental pots (24 cm diameter, 45 cm height) were filled with soils collected from the 0–30 cm layer from a maize (*Zea mays* L.) cultivation area.

All the fertilizers were added as basal fertilization before planting maize. Seeds of maize were germinated on moist filter paper for 2 d, and seedlings were then planted in 90 pots (two plants per pot, 15 pots per treatment). The pots were located in a micro-region in the cropland at the study site. Urea (N = 46%) was chosen as the N treatment source. The Technology University of South China provided a bacterial fertilizer (HYSD001, Guangzhou Huayuan Biotechnology Company) that contained a variety of phosphorus-solubilizing and nitrogen-fixing bacteria as exogenous microorganisms. Cattle compost (M), which was fermented for three months (June to September) under high temperatures, was collected from the Hongyi Organic Farm in the village. According to the nitrogen fertilizer requirement (150 kg·hm^−2^) for maize growth, equal amounts of N in different treatments except for the B treatment were applied. Based on the 60% water content of cattle compost, the quantity of cattle compost (pH was 7.2; organic matter content was 440.43 g·kg^−1^; C/N was 26.7; nitrogen content was 1.6%) in the M and MB treatments was 3.75 kg·m^−2^, the quantity of urea (nitrogen content was 46%) in the N and NB treatments was 32.6 g·m^−2^, and the quantity of bacterial fertilizer in the B, MB, and NB treatments was 1.56 g·m^−2^. A conventional management scheme in China was conducted for pest and weed management during the experiment [21].

### Sampling and processing

Soil samples were collected in triplicate at the maize seeding, tasseling, and mature stages from a 0–20 cm depth in each pot. Each soil sample was separated into two parts. One part was air-dried and stored at room temperature for determining soil chemical properties. The other part was passed through a 2-mm sieve, moistened to 60% of their water holding capacity, and immediately stored at 4°C for the measurement of soil microbial properties.

### Soil microbial biomass and respiration

Microbial biomass C (MBC) and biomass N (MBN) contents were estimated by using chloroform fumigation extraction [22]. The soil samples were divided into two portions. One 15-g portion (in dry weight) rewetted to 60% was fumigated for 24 h at 25°C with chloroform (ethanol-free). After fumigant removal, the soils were extracted with 60 mL of 0.5 mol·L^−1^ K_2_SO_4_ and placed on a horizontal shaker at a speed of 300 r·min^−1^ for 30 min and filtered. The non-fumigated portion was extracted similarly as fumigation commenced. Total dissolved N and C in the K_2_SO_4_ extracts were determined by using an automated C analyzer (Shimadzu, TOC-VCPH, Japan). Regarding the incomplete extraction, two conversion factors were applied to calculate the biomass, as 0.54 for MBN (*k_EN_*) and 0.45for MBC (*k_EC_*) [23] in the following equation (1) and (2).
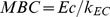
(1)

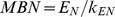
(2)where *E_C_* refers to the different organic carbon amount between fumigated and non-fumigated treatment, and *E_N_* represents the difference in total nitrogen [24], [25].

Basal soil respiration was determined by using the sealed jar incubation method, which employed a trap of 0.5 mol of NaOH alkali CO_2_ [26]. At sampling, the jar lid was opened, the alkali trap was removed, and the solution was back-titrated with 0.5 mol HCl in order to assess CO_2_ release. The alkali trap was replaced during each measurement.

### Plate counts of cultivable microorganisms

The total number of cultivable bacteria, fungi, and actinomyces were counted as colony forming units (CFUs) on agar plates using the dilution plate method. The media used for the enumeration of bacteria, fungi, and actinomyces were beef extract peptone medium, Czapek's medium, and Gause's No. 1 synthetic medium, respectively [27].

### DNA extraction and DGGE

To determine soil microbial diversity, we used the molecular biology methods of DGGE. Briefly, soil DNA was extracted by using the Power Soil DNA Extraction Kit (Mobio Laboratories), following the manufacturer's instructions. For bacterial community analysis, the V3 region of 16S rRNA gene were amplified by touchdown polymerase chain reaction (PCR) using two different primer sets, 518R (5′-ATT ACC GCG GCT GCT GG) and GC-338F (5′-CGC CCG CCG CGC GCG GCG GGC GGG GCG GGG GCA CGG GGG GCC TAC GGG AGG CAG CAG), containing the GC clamp on the amplified 16S rDNA template. For fungal community analysis, the fragments of 18S rRNA gene were amplified by nested PCR using the primer sets GC-fung (5′-CGC CCG CCG CGC CCC GCG CCC GGC CCG CCG CCC CCG CCCCATTCCCCGTTACCCGTTG-3′) and NS1 (5′-GTAGTCATATGCTTGTCTC-3′).

PCR mixtures containing 25 µL of Premix Taq (Takara Biotechnologies), 1.5 µL of each primer, and 2 µL of the DNA template were made up to a volume of 50 µL with sterile Milli-Q water. The samples were amplified in a Peltier Thermal Cycler (PTC-200) (Bio-Rad Laboratories, Hercules, CA). The amplification conditions of bacteria and fungi samples were as follows: an initial denaturation of DNA for 5 min at 94°C, followed by 25 cycles of 30 s at 94°C, 30 s at 55°C, and 30 s at 72°C; 10 cycles of 30 s at 92°C, 30 s at 55°C, and 45 s at 72°C; and a final extension at 72°C for 10 min. Blank controls were used through all the steps.

DGGE was performed with a DCode universal mutation detection system (Bio-Rad Laboratories, Hercules, CA). Approximately 1 µg of the bacteria PCR product per lane were loaded onto 8% polyacrylamide (37.5∶1 acrylamide: bisacrylamide) gels in a 1× TAE buffer with a bacteria denaturing gradient ranging from 40% to 60%, whereas that for fungi ranged from 15% to 40%. Gel electrophoresis was performed at 60°C for 13 h at 80 V. The gel was subsequently stained in a 1× TAE buffer containing 1∶10000 dilution of SYBR Green I nucleic acid staining solution (GenScript, USA) for half an hour before being photographed on a Molecular Imager Gel Doc XR System (Bio-Rad Laboratories).

The soil microbial community diversity indexes were calculated in the following equation (3), (4) and (5):
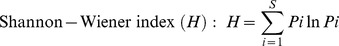
(3)


Where: *Pi* is the ratio of the activity on a particular substrate to the sum of activities on all substrates; *S* is the sum of band number in one sample.

Evenness index (*E*):
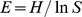
(4)


Where: *H* – Shannon index; *S* - band numbers of every substrate.

Simpson index (*C*):
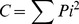
(5)


Where: *Pi* is the ratio of the activity on a particular substrate to the sum of activities on all substrates.

### Soil enzyme activity

Urease activity was determined by using the phenol-sodium hypochlorite colorimetric method [28], as indicated by mass (mg) of NH_3_-N in 1 g soil incubated for 24 h (U). Catalase activity was analyzed by using the titration method [29], and its activity was indicated by depletion (mL) of KMnO_4_ (0.1 mol L^−1^) after 20 min of 1 g incubated soil. Invertase activity was determined by using the 3,5-dinitrosalicylate colorimetric method [24], which was indicated by the mass (mg) of glucose of 1 g soil after 24 h. Cellulase activity was measured by using the 3,5-dinitrosalicylate colorimetric method [29], which was indicated by the mass (mg) of decomposed generated glucose (mg) from cellulose of 1 g soil after 72 h.

### Statistical analysis

All statistical analyses were carried out using the SPSS 16.0 software. A two-way ANOVA at the 0.05 level was conducted to determine the interactions between fertilizer treatments and maize stages. And one-way ANOVA was used for analysis of significant difference among fertilizer treatments and plant stages.

## Results

### Soil microbial biomass and soil respiration rate

**Figure 1:**
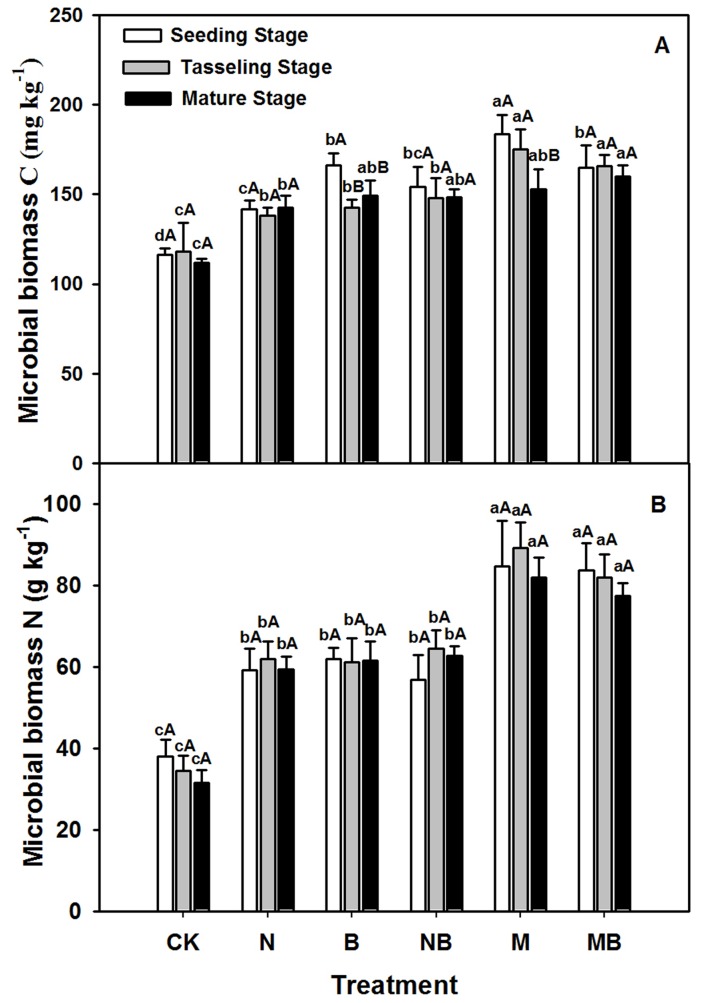
Soil microbial biomass C and soil microbial biomass N in various treatments during maize growth stages. CK: no fertilizer; N: N fertilizer; B: bacteria fertilizer; NB: N fertilizer + bacteria fertilizer; M: manure compost; MB: manure compost + bacteria fertilizer. Data are means ± SD (n = 3). Different lower case letters indicate significant differences (ANOVA, LSD test, *p*<0.05) among fertilizer treatments; different upper case letters indicate significant differences among maize growth stages.

Soil MBC and MBN over time in different treatments are presented in Fig. 1. For all sampling times, MBC significantly increased in the fertilization treatments against their controls (CK) (*p*<0.01) during the whole maize growth stage, with the largest increment of MBC occurring consistently in the MB treatment. Differences between the MB treatment and other treatments except for the M treatment reached significance at the tasseling stage (*p*<0.01). The ANOVA results showed that MBC in the M and MB treatments were significantly higher than those in the B (*p*<0.05), N (*p*<0.01) and NB treatments at the maize tasseling stage (*p*<0.01). However, no significant differences were noted between MB and M or between N and NB treatments (Fig. 1A). The fertilizers also affected soil MBN, which varied among different treatments. Compared with the CK treatment, all fertilizer treatments showed significantly higher MBN, and this was greater in the M and MB treatments than in the N and NB or B treatments (*p*<0.01). Nevertheless, no statistically significant differences (*p*>0.05) were observed among the B, N, and NB treatments.

**Figure 2:**
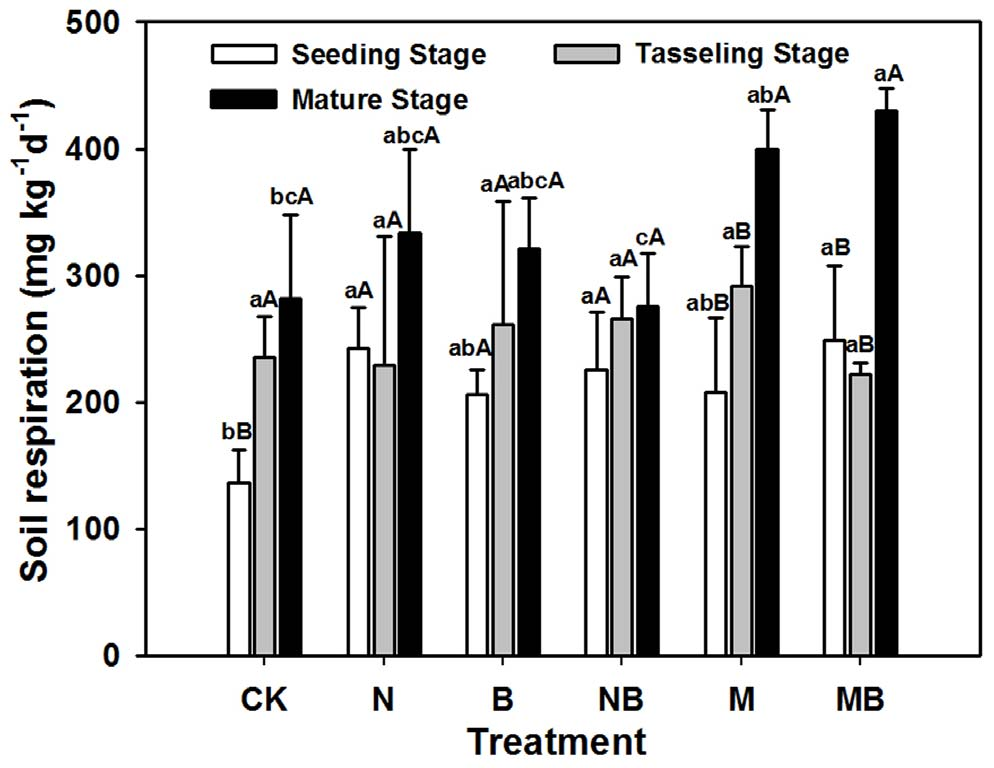
Soil respirations rate in various treatments during maize growth stages. CK: no fertilizer; N: N fertilizer; B: bacteria fertilizer; NB: N fertilizer + bacteria fertilizer; M: manure compost; MB: manure compost + bacteria fertilizer. Data are means ± SD (n = 3). Different lower case letters indicate significant differences (ANOVA, LSD test, *p*<0.05) among fertilizer treatments; different upper case letters indicate significant differences among maize growth stages.

Soils with different fertilizers showed higher respiration rates and greater cumulative CO_2_ production compared to those observed in the CK treatment (Fig. 2). A significantly enhanced soil respiration rate was observed during the entire period. Compared with other fertilizer treatments, only M and MB treatments showed significant higher respiration rates at mature stage than seeding and tasseling stages. MB treatment exhibited drastically higher soil respiration rate than those of the NB and N treatments at the mature stage (*p*<0.01), whereas M treatment was only higher than NB treatment. However, soils with N and B treatments only showed higher respiration rates than those of the NB treatment, indicating that fertilizers could significantly enhance the microbial activities, and the highest number and greatest effects were observed from organic amendments.

### Plate counts of cultivable microorganisms

**Figure 3:**
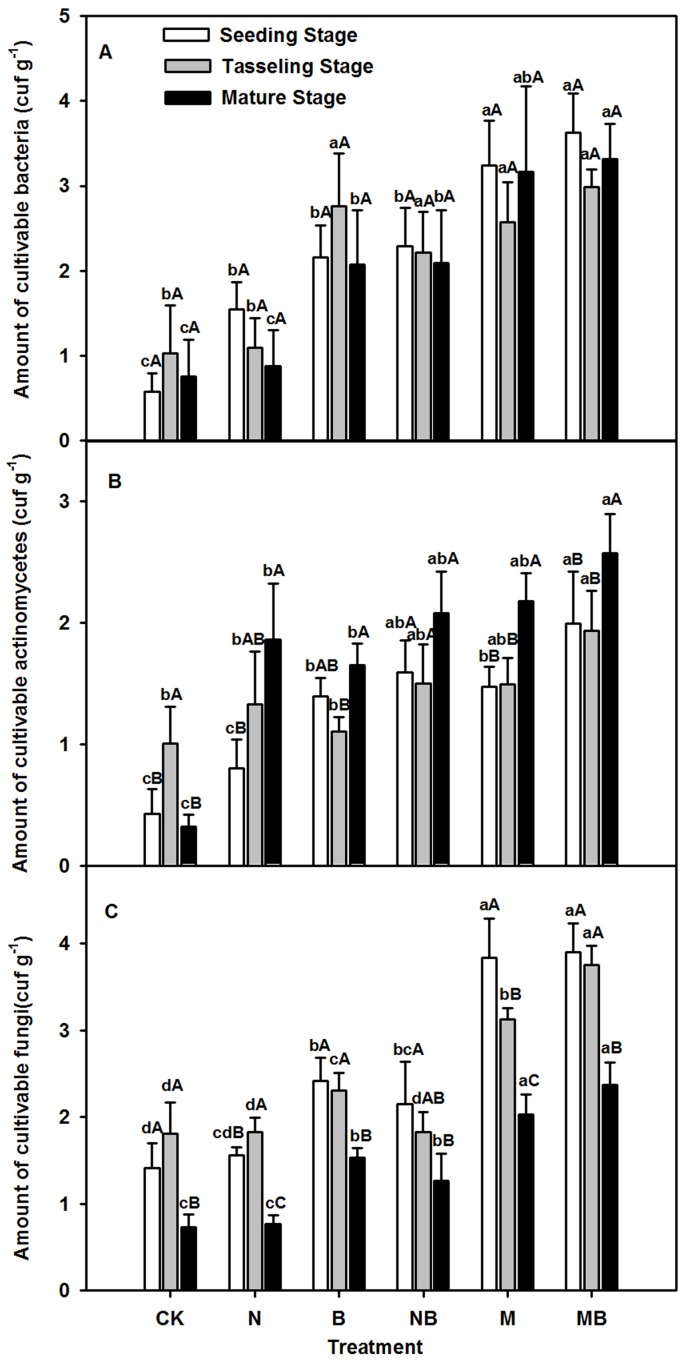
The clony-forming units of bacteria, fungi and actinomyces in various treatments during maize growth stages. CK: no fertilizer; N: N fertilizer; B: bacteria fertilizer; NB: N fertilizer + bacteria fertilizer; M: manure compost; MB: manure compost + bacteria fertilizer. Data are means ± SD (n = 3). Different lower case letters indicate significant differences (ANOVA, LSD test, *p*<0.05) among fertilizer treatments; different upper case letters indicate significant differences among maize growth stages.

There were extremely large variations in the number of bacteria, fungi, and actinomyces among various treatments (Fig. 3). The colony-forming units (CFUs) of bacteria in all fertilizer treatments (except N treatment) were significantly higher than those observed in the CK treatment (*p*<0.01) at the tasseling and mature stages (Fig. 3A). At the mature stage, the number of CFUs of bacteria in the M and MB treatments were significantly higher than that in the N treatment (*p*<0.01), with the bacterial numbers from the MB treatment considerably higher than those observed in the B and NB treatments (*p*<0.05) (Fig. 3A). However, the number of bacteria in the M treatment did not differ from that in the B and NB treatments (*p*>0.05). Fertilizers also largely influenced the amount of soil fungi. For instance, the amount of fungi in all fertilizer treatments was remarkably higher than that in the CK treatment (*p*<0.01) at the mature stage (Fig. 3B). At the same stage, the amount of fungi in the MB treatment was significantly higher than that in the B (*p*<0.01) and N (*p*<0.05) treatments, however this was not the case in the M and NB treatments. And the amount of fungi in all the fertilizer treatments (expect NB treatment) largely increased at mature stage. Similar to bacteria and fungi, the number of cultivable actinomyces was also affected. The amount of cultivable actinomyces in the M and MB treatments was considerably higher than that in the B and NB treatments, both were greatly higher than that observed in the CK and N treatments (*p*<0.01) (Fig. 3C). Meanwhile, the amount of cultivable actinomyces in the CK and N treatments initially increased, but eventually decreased at the end of growth. In the B, NB, M, and MB treatments, cultivable actinomyces decreased during the entire maize growth period.

### Soil microbial community composition structure

**Figure 4:**
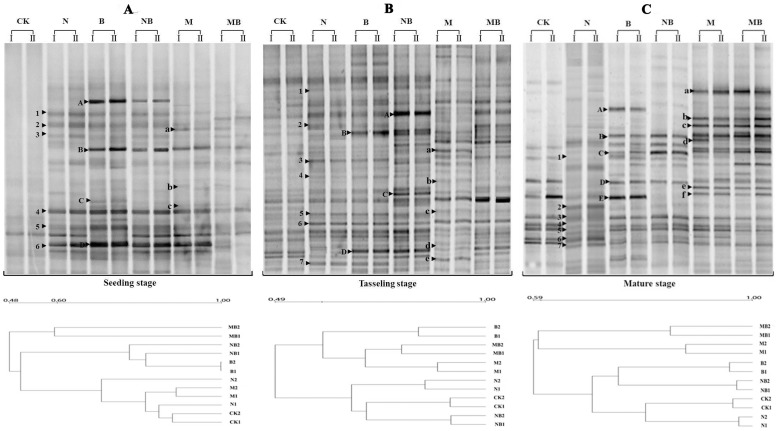
DGGE banding patterns of 16S bacteria fragment and clustering of DGGE profiles in various treatments during maize growth stages. CK: no fertilizer; N: N fertilizer; B: bacteria fertilizer; NB: N fertilizer + bacteria fertilizer; M: manure compost; MB: manure compost + bacteria fertilizer.

Changes in the microbial community structure were determined by PCR-DGGE analysis, which targeted domains of bacteria and fungi. As shown in Fig. 4, different fertilizers significantly affected the community structure of soil bacteria and fungi at all sampling times. DGGE bands that could be enhanced by all fertilizers were marked with numbers. Bands enhanced by the N or NB treatments were marked with uppercase alphabet letters, whereas those enhanced by the M or MB treatments were marked with lower case alphabet letters (Fig. 4).

For bacterial community structure, all the fertilizers stimulated the generation of band numbers 1–6. N, B or NB treatments stimulated the occurrence of bands A–-D, whereas M or MB enhanced the bands a–c at the seeding stage. The influences from N, B or NB treatment on bacterial community structure were lower than those from M or MB treatment (Fig. 4). The number of DGGE bands was the highest at the tasseling stage, indicating that the formed microbial community was the most diverse at this stage. We therefore confirmed that the largest influences of bacteria community structure occurred after the fertilizer treatments of M and MB. The results at the mature stage were similar to the tasseling stage, *e.g.*, the influences on bacterial community structure from the M or MB treatments were higher than that of the N or NB treatments (Fig. 4B and 4C). Furthermore, statistical analysis showed that manure compost and bacterial fertilizer could enhance the Shannon index, whereas the N and NB treatment decreased the Shannon index, especially from seeding stage to tasseling stage (Table 1). The Shannon indexes of the M or MB treatments were higher than those of the other treatments, which remained high during the entire experiment except seeding stage (Table 1). At tasseling stage the Shannon indexes of the N, NB, M and MB treatments significantly higher than CK and N treatments. The results at the mature stage were similar to the tasseling stage, the Shannon indexes of M and MB treatments were higher than other fertilizer treatments. Although the Shannon index in the N treatment was the lowest at tasseling and mature stage, a downtrend trend was observed. The evenness indexes (except seeding stage) and the Simpson indexes in all treatments were basically the same, and retained a smooth and steady trend during the entire growth period, and there was no significant difference among maize growth stages (Table 1).

**Table 1:** Effect of different treatments on soil bacterial community structure diversity as evaluated by Shannon index (*H*), **Evenness** index (*E*) and Simpson index (*C*) in the DGGE-PCR.

Treatments	Shannon-Wiener (*H*)			Evenness index (*E*)			Simpson index (*C*)		
	Seeding	Tasseling	Mature	Seeding	Tasseling	Mature	Seeding	Tasseling	Mature
CK	2.16±0.25cB	2.62±0.01cA	2.53±0.06cA	0.96±0.01bA	0.98±0.01aA	0.95±0.01aA	0.07±0.03aA	0.05±0.01aA	0.06±0.01aA
N	2.84±0.03abA	2.69±0.04cA	2.40±0.05cB	0.96±0.01bA	0.98±0.01aA	0.98±0.01aA	0.05±0.01aA	0.05±0.01aA	0.05±0.01aA
B	2.78±0.02abA	2.82±0.01abA	2.76±0.21bA	0.97±0.01abA	0.98±0.01aA	0.97±0.01aA	0.06±0.01aA	0.05±0.01aA	0.06±0.01aA
NB	2.92±0.04aA	2.86±0.08abA	2.83±0.13bA	0.98±0.01abA	0.96±0.01aA	0.98±0.01aA	0.05±0.01aA	0.05±0.01aA	0.06±0.01aA
M	2.54±0.04bB	3.23±0.05aA	3.44±0.03aA	1.00±0.01aA	0.96±0.01aB	0.96±0.01aB	0.07±0.01aA	0.05±0.01aA	0.05±0.01aA
MB	2.60±0.12bB	3.31±0.06aA	3.41±0.11aA	0.96±0.01bA	0.97±0.01aA	0.96±0.01aA	0.09±0.01aA	0.06±0.01aA	0.05±0.01aA

Data analyzed by two-way ANOVA, LSD test, *p*<0.05. Different lower case letters indicate significant difference among fertilizer treatments; different upper case letters indicate significant difference among maize growth stages.

Cluster analysis showed that bacterial community structures in treatments B and NB had a higher degree of similarity, while N, M and CK got closed and MB forms a separate category at the seeding stage (Fig. 4A). M and MB got together and B formed a separate category, whereas CK, NB and N are in another group at the tasseling stage (Fig. 4B). At the mature stage, M and MB had a higher degree of similarity, while other treatments classified to a similar category (Fig. 4C).

**Figure 5:**
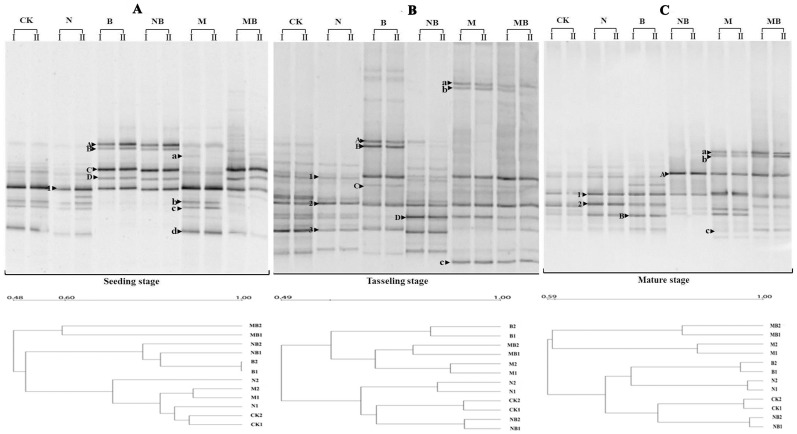
DGGE banding patterns of 18S rDNA fungi fragment and clustering of DGGE profiles in various treatments during maize growth stages. CK: no fertilizer; N: N fertilizer; B: bacteria fertilizer; NB: N fertilizer + bacteria fertilizer; M: manure compost; MB: manure compost + bacteria fertilizer.

In terms of fungal community structure, the number of DGGE bands was highest at tasseling stage, and the largest influences on fungi community structure occurred with the M and MB treatments (Fig. 5). Statistical analysis showed that manure compost could enhance the Shannon index, whereas the N treatment decreased the Shannon index (Table 2). At seeding and tasseling stages, the Shannon index of the MB treatment appreciably higher than other treatments except M treatment, and the Shannon index of the M treatment was the highest at mature stage. However, the Shannon index of the N treatment rapidly decreased during the entire growth period, which was the lowest at tasseling and mature stages. The evenness index in all fertilizer treatments had no significant difference. The simpson index of M and MB treatments were notably lower than those of N, B and NB treatments during the entire experiment (Table 2). Cluster analysis of fungal community structure also showed that fungal community structure in treatments added bacterial fertilizer (B, NB and MB) were similar at the initial seeding stage, while other treatments (CK, N and M) formed another separate category (Fig. 5). M and MB become more and more similar with time, while CK, N and M treatments were in another group, and B forms a separate category at the tasseling and mature stages (Fig. 5).

**Table 2:** Effect of different treatments on soil fungi community structure diversity as evaluated by Shannon index (*H*), **Evenness** index (*E*) and Simpson index (*C*) in the DGGE-PCR.

Treatments	Shannon-Wiener (*H*)			Evenness index (*E*)			Simpson index (*C*)		
	Seeding	Tasseling	Mature	Seeding	Tasseling	Mature	Seeding	Tasseling	Mature
CK	1.86±0.18cA	2.08±0.01cA	1.04±0.01cB	0.96±0.01aA	0.96±0.01aA	0.95±0.01aA	0.12±0.03cB	0.09±0.01cB	0.18±0.01aA
N	2.03±0.11bA	1.81±0.12dA	0.69±0.01dB	0.93±0.01aA	0.96±0.01aA	0.98±0.01aA	0.15±0.01cA	0.17±0.02bA	0.19±0.01aA
B	1.83±0.16cB	2.55±0.04bA	1.06±0.01cC	0.95±0.02aA	0.98±0.01aA	0.97±0.01aA	0.24±0.02aB	0.29±0.01aA	0.17±0.01aC
NB	1.84±0.17cB	2.26±0.04cA	1.03±0.01cC	0.95±0.01aA	0.96±0.01aA	0.94±0.01aA	0.19±0.03bB	0.25±0.01aA	0.20±0.01aB
M	2.13±0.03abB	2.65±0.11abA	2.71±0.01aA	0.93±0.01aA	0.98±0.01aA	0.96±0.01aA	0.13±0.01cA	0.11±0.01cA	0.15±0.01bA
MB	2.20±0.02aB	2.79±0.08aA	2.55±0.20bA	0.96±0.01aA	0.98±0.02aA	0.96±0.034aA	0.12±0.01cA	0.08±0.005cB	0.14±0.06bA

Data analyzed by two-way ANOVA, LSD test, *p*<0.05. Different lower case letters indicate significant difference among fertilizer treatments; different upper case letters indicate significant difference among maize growth stages.

The results of bacteria and fungi community structure analysis indicated that organic and chemical fertilizers could enhance the bacteria and fungi community by enhancing the microbial diversity at the tasseling and mature stages, while bacterial fertilizer significantly influenced the bacteria and fungi community in initial seeding stage (Table 2).

### Soil enzyme activities

**Figure 6:**
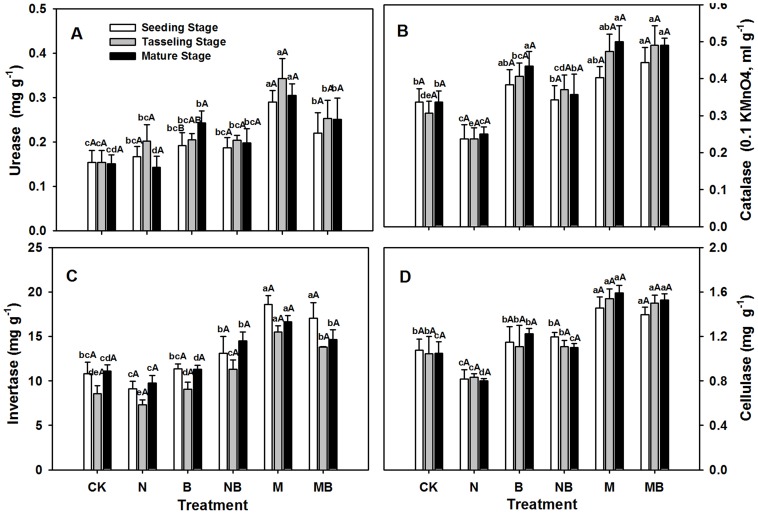
Soil enzyme activity in various treatments during maize growth stages. CK: no fertilizer; N: N fertilizer; B: bacteria fertilizer; NB: N fertilizer + bacteria fertilizer; M: manure compost; MB: manure compost + bacteria fertilizer. Data are means ± SD (n = 3). Different lower case letters indicate significant differences (ANOVA, LSD test, *p*<0.05) among fertilizer treatments; different upper case letters indicate significant differences among maize growth stages.

Urease activity in M and MB treatments were higher than that in the CK treatment (*p*<0.01), especially in the M treatment (Fig. 6). No significant differences were observed between the N and CK treatments at the seeding, tasseling stages and mature stages. However, urease activity in the N treatment rapidly decreased in the mature stage of maize (Fig. 6A). However, the catalase activities in the M and MB treatments were significantly higher than those observed in the CK treatment at tasseling and mature stages (*p*<0.01), and it was similar for B treatment at the mature stage. On the other hand, the activity of catalase in the N treatment was significantly lower than that in the CK treatment at all sampling times (*p*<0.05) (Fig. 6B).

Invertase activity in the M and MB treatments was appreciably higher than that in the CK treatment during the entire growth period (*p*<0.01), although no differences were observed between the B and CK treatments. N treatment showed the lowest invertase activity at all times, although this was not statistically different from that observed in the CK treatment (Fig. 6C). The M and MB treatments showed a significantly enhanced cellulase activity (*p*<0.01), whereas that in the N treatment decreased (*p*<0.01). No big differences in cellulase activity were observed among the NB, B, and CK treatments, indicating that the influence of bacterial fertilizer on cellulase activity was not significant (Fig. 6D). These results suggest that manure compost was better than bacteria fertilizer alone in enhancing soil enzyme activities, whereas chemical fertilizers degrade the activities of soil enzymes.

### Soil physicochemical and chemical properties

The humus content in the M and MB treatments was remarkably higher than that in the B and CK treatments, whereas humus content in the N treatment was significantly lower than that in the CK treatment. Available N in the M and MB treatments were also considerably higher than that in the CK treatment, whereas there was no difference between the N and CK treatments. Fertilizers showed minimal impact on soil pH, total N, and available P during the entire maize growth period. Positive correlations were observed between soil organic matter (SOM) and microbial biomass (MBC and MBN), humus and microbial biomass (MBC and MBN), SOM and enzymes activities, humus and enzymes activities (urease, catalase, invertase, and cellulase) (Table 3), whereas the correlations among available N, microbial biomass, and enzymes activities were not significant. The addition of fertilizers enriched the soil microbial biomass and soil enzymes by enhancing the soil physicochemical properties of SOM and humus, especially through the addition of manure compost.

**Table 3:** Correlation of MBC, MBN, Available N, SOM, Humus and enzymes activities.

Variables	MBC	MBN	Available N	SOM	Humus	Urease	Catalase	Invertase
MBN	0.910**							
Available N	0.425	0.518*						
SOM	0.777**	0.820**	0.380					
Humus	0.685**	0.750**	0.621**	0.622**				
Urease	0.721**	0.694**	0.232	0.558*	0.826**			
Catalase	0.585*	0.620**	0.410	0.646**	0.871**	0.835**		
Invertase	0.582*	0.687**	0.238	0.580*	0.776**	0.732**	0.703**	
Cellulase	0.615**	0.663**	0.528*	0.614**	0.948**	0.838**	0.928**	0.790**

*, Pearson correlation is significant at the 0.05 level.

**, Pearson correlation is significant at the 0.01 level.

### PCA combined with a hierarchical clustering analysis

**Figure 7:**
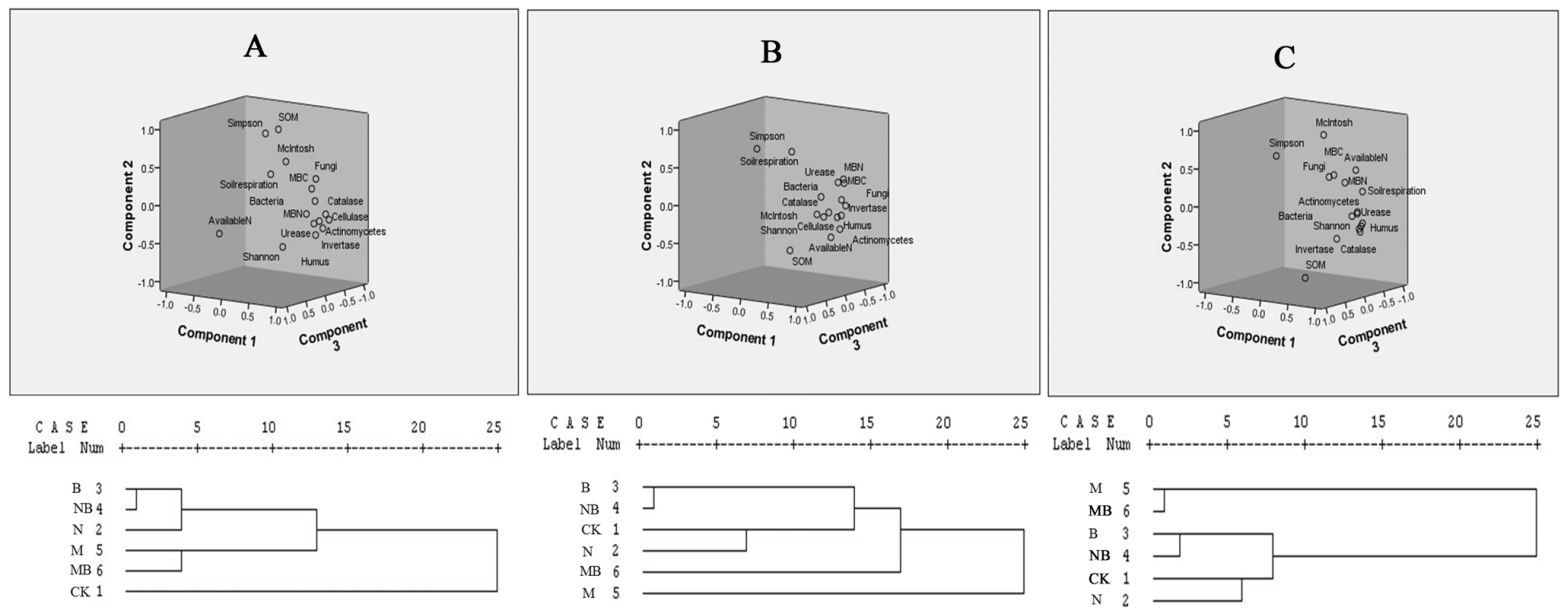
PCA of indexes combined with a hierarchical clustering analysis in various treatments during maize growth stages. CK: no fertilizer; N: N fertilizer; B: bacteria fertilizer; NB: N fertilizer + bacteria fertilizer; M: manure compost; MB: manure compost + bacteria fertilizer. A: Seeding stage; B: Tasseling stage; C: Mature stage.

Kaise-Meyer-Olkin Measure showed the KOM value of three phases is 0.798>0.7, and the F value is 0 with Bartlett's Test of Sphericity test. All data obey the normal distribution, and are suitable for principal component analysis (Fig.7).

At seeding stage, three factors were extracted as the main composition factor, and the cumulative variance contribution rate was 93.49 (Fig.7A). The first principal component contribution rate was the largest, accounting for 59.94, and the main relevant parameters were soil microbial indicators (bacteria, fungi, actinomycetes, MBN, MBC), enzyme activities (invertase urease and cellulase) and humus. The main relevant parameters of second and third principal component were SOM and Available N, accounting for 20.06 and 13.49, respectively. According to the selection indexes of principal component, the ahierarchical clustering analysis was used and the result showed that treatments of N, B and NB form a separate category, while M and MB were in another group, and CK formed a separate category (Fig.7A). At tasseling stage, the cumulative variance contribution rate of three principal components factors was 90.12. The first principal component contribution rate was 67.68, with the main relevant parameters of soil microbial indicators (actinomycetes,Shannon, bacteri, MBN and MBC), enzyme activities (Cellulas, Catalase and Invertase) and humus (Fig.7B). The main relevant parameters of second and third principal component were SOM and simpson, accounting for 12.39 and 10.05, respectively. Cluster analysis showed that N, B, NB and CK had a higher degree of similarity, whereas M and MB formed a separate category, respectively (Fig.7B). At mature stage, the cumulative variance contribution rate was 94.45. The variance contribution of the first principal components was 64.154, and the mainly relevant parameters were soil microbial indicators (bacteria, actinomycetes, shannon and MBN), enzyme activities (cellulas and catalase) and humus. The variance contribution of the second principal components was SOM with 20.21. The variance contribution of the third principal components was simpson index, accounting for 10.093. Hierarchical clustering analysis showed that M and MB had a higher degree of similarity and treatments of B and NB forms a category, while CK and N are in another group (Fig.7C).

## Discussion

### Soil microbial community structure

In cropland soils, the application of fertilizers imparted a stronger effect on microbial communities, as shown by previous studies involving the analysis of PLFA, microbial biomass, and rRNA gene libraries [20]. DGGE profiles revealed that the band numbers in all fertilizer treatments significantly increased at various maize growth stages, with band numbers in M and MB treatments higher than those observed in the other fertilizer treatments (B, NB, and N) (Figs. 4 and 5). Our results suggest that cattle compost changes the soil bacteria and fungi community structure. Previous investigations have also demonstrated that animal compost increased bacterial and fungi diversity by increasing the carbon pool of the soil, thus improving the living conditions for indigenous microbial populations [30], [31]. Our study showed that soil organic matter and humus in the M treatment were the highest. The DGGE profiles (Figs. 4C and Figs. 5B) and Shannon index data showed that the bacterial and fungi diversity of the M and MB treatments remained stable, whereas these declined in the N, B, and NB treatments at maize mature stage.

The M treatment resulted in an increase in exogenous microorganisms, which can directly influence the soil microorganism community structure [32]. The specific bands in the DGGE profiles showed that exogenous microorganisms emerged in the M and MB treatments, thus clearly supporting this statement (Figs. 4 and 5). The M treatment greatly improved microbial diversity, indicating that M is conducive to the establishment of a diverse microbial community structure (Tables 1 and 2).

The application of bacterial fertilizer provided a variety of nitrogen-fixing bacteria and phosphorus-solubilizing bacteria that could improve the soil microbial community structure (Figs. 4 and 5). New fungal species, which may act as indicators of exogenous microorganism, were thus introduced (Fig. 5). The Simpson index of soil fungi community in the MB treatment was lowest during the entire stages, and B and NB treatments was the highest respectively at seeding stage and last two stages (Table 2), suggesting that exogenous microorganisms played a dominant role in both stages, which combined with compost enhanced soil microbial community uniformity [33],while N fertilizer always displayed less or negative influences compared with the other treatments. For example, the Shannon index of soil bacterial and fungal communities showed that the soil microbial community diversity in the N and NB treatment gradually decreased compared to that in the B treatment, displaying an obvious negative influence from the administration of nitrogen fertilizers (Tables 1 and 2). This may be attributable to the absence of an external carbon source [34]. Nevertheless, only adding N fertilizers may inhibit the soil microorganism propagation rather than increase it, which may further degrade the bacteria and fungi community diversity [35].

### Soil microbial biomass and soil activity

Soil microbial biomass is an important source of plant nutrients and is highly correlated with SOC (soil organic carbon) [34]. Enhancement of soil microbial activity was associated with high soil available N for plants [36]. The M and MB treatments significantly increased soil MBC, MBN, and respiration rates, which positively influenced microbial processes and development. These findings also showed that M significantly enhanced the density of soil bacteria, fungi, and actinomycetes. These indexes were significantly positively correlated with humus (*p*<0.05) (Table 3), indicating that the M treatment served as a sufficient carbon source that enhanced the microorganism biomass and activity.

Microbial carbon and nitrogen initially increased and then subsequently decreased during the maize growth period, achieving the highest value at the tasseling stage. This finding might be attributable to the competition of carbon and nitrogen source between soil microorganisms and crops. With the transformation of soil nutrients, the competitiveness of crops is enhanced, thus leading to a decrease in microbial carbon and nitrogen [37].

### Effects on soil enzymes

Soil microbial enzymes are mainly driven by metabolic processes, largely reflecting the level of soil microbial activity and the intensity of biochemical reactions [38]. Our investigation demonstrated that different fertilizers have significantly affected soil enzyme activities (*p*<0.01) (Fig. 6). Four kinds of soil enzyme activities consistently displayed the highest levels in the M or MB treatments (Fig. 6). The M treatment can accelerate soil carbon and nitrogen circulation [14] and improve soil quality, with enzyme activities stemming from manure compost. As manure compost contains abundant organic matter, it can drastically increase SOM by providing a rich source of carbon and nutrients for enzyme production microorganisms.

However, the application of N fertilizer had less influence on soil urease activity, whereas it significantly decreased soil catalase, invertase, and cellulase activities (*p*<0.01) (Fig. 6). It is thus possible that the application of chemical fertilizers has inhibited enzyme production in microorganisms. Except for urease activity, the B and NB treatments showed no influence on the activities of the other three enzymes (Fig. 6), suggesting that NB can enhance the soil nitrogen transformation.

### Improvement of soil fertility

The N treatment notably reduced microbial biomass and enzyme activities compared to those with the other treatments. This finding could be attributable to the acidifying effect of long-term N fertilizers, as showed by lower pH in previous studies [39]. However, no significant differences in soil pH were observed between most fertilizers and the CK treatment, which maintained a neutral pH during the maize growth period. It is possible that the acidifying effect of the N fertilizer may have not been reflected within a relatively short period. SOC is a pivotal component of soil fertility, and the level of organic matter is influenced by the application of fertilization [40]. In a healthy soil, the level of SOC is considered to be a functional indicator of the net input of organic residues [41]. In our study, manure compost enhanced the accumulation of SOC, which is consistent with the findings of several other previous studies from many countries [42], [43]. The improved SOC may be due to a higher humectation rate and a constantly lower decay rate, since SOC is a nutrition pool that maintains microbial activity. SOC, especially humus, was significantly positively correlated with soil microbial biomass and soil enzyme activity (Table 3). However, there was no obvious change in the total N level under manure compost, which may partially be due to a slow release of N from manure compost and smaller losses of N as described by Bhandari et al. [42].

The results of PCA combined with a hierarchical clustering analysis showed that fertilizer treatments had been divided into three groups at the end stage (Fig.7). The application of manure compost treatments (M and MB) had a higher degree of similarity; the application of bacterial fertilizer bacterial fertilizer treatments (B and NB) formed another category, while treatments of CK and N are in another group. Compared to the application of bacterial fertilizer treatments, the distance of application of manure compost treatments was far away with the group of CK and N. These result exactly demonstrated that the application of manure compost has more influences on soil microbial community diversity and soil microenvironments than bacterial fertilizer.

## Conclusions

Manure compost, especially manure compost + bacterial fertilizer, consistently resulted in higher levels of soil respiration rate, cultivable microorganisms, and soil enzyme activities, while N fertilizers showed no significant influence or negative results. The number of DGGE bands with bacteria and fungi also indicated that the Shannon index of manure compost treatment was the highest and remained high. Fertilizers, especially manure compost, significantly enhanced soil microbial properties in response to the increase in soil physicochemical properties of soil organic matter and humus. From a soil microbial point of views, manure compost application can be used as an environmentally friendly and rapid measure for restoring degraded cropland.
